# The role of MRI-R2* in the detection of subclinical pancreatic iron loading among transfusion-dependent sickle cell disease patients and correlation with hepatic and cardiac iron loading

**DOI:** 10.1186/s13244-022-01280-x

**Published:** 2022-09-04

**Authors:** Basant Mohamed Raief Mosaad, Ahmed Samir Ibrahim, Mohamed G. Mansour, Mohsen Saleh ElAlfy, Fatma Soliman Elsayed Ebeid, Emad H. Abdeldayem

**Affiliations:** 1 Radiology Department, Faculty of Medicine, Ain Shams University, Cairo, Egypt; 2 Pediatrics Department, Faculty of Medicine, Ain Shams University, Cairo, Egypt

**Keywords:** Pancreatic R2*, Sickle cell disease, MRI T2*

## Abstract

**Objectives:**

Pancreatic reserve could be preserved by early assessment of pancreatic iron overload among transfusion-dependent sickle cell disease (SCD) patients. This study aimed to measure pancreatic iron load and correlate its value with patients’ laboratory and radiological markers of iron overload.

**Materials and methods:**

Sixty-six SCD children and young adults underwent MRI T2* relaxometry using a simple mathematical spreadsheet and laboratory assessment.

**Results:**

The results indicated moderate-to-severe hepatic iron overload among 65.2% of studied cases. None had cardiac iron overload. Normal-to-mild iron overload was present in the pancreas in 86% of cases, and 50% had elevated serum ferritin > 2500 ug/L. There was no significant correlation between pancreatic R2* level, serum ferritin, and hepatic iron overload. Patients with higher levels of hemolysis markers and lower pre-transfusion hemoglobin levels showed moderate-to-severe pancreatic iron overload.

**Conclusion:**

Chronically transfused patients with SCD have a high frequency of iron overload complications including pancreatic iron deposition, thereby necessitating proper monitoring of the body’s overall iron balance as well as detection of extrahepatic iron depositions.

## Key points

- Transfusion-dependent SCD patients did not demonstrate cardiac siderosis.
- A total of 86% of transfusion-dependent SCD patients had normal-to-mild pancreatic iron overload.
- No correlations were found between pancreatic R2* level, transfused iron, or hepatic iron.

## Introduction

Sickle cell disease (SCD) is an autosomal recessive hematological disorder involving production of abnormal sickle hemoglobin (HbS) [1]. The responsible gene exists in the Egyptian western desert near the Libyan border with variable rates of 0.38% in coastal areas and up to 9.0% in the new valley oases, mostly of the African globin gene haplotype [2].

Although wider use of hydroxycarbamide and new therapeutic approaches have improved health-related quality of life, SCD in lower-resource countries still carries a poor prognosis and is associated with high early childhood mortality [3]. Transfusion is a frequently employed therapy that is best validated for prophylaxis and treatment of stroke, preoperative prophylaxis, and treatment of acute chest syndrome (ACS) [4]; about 90% of adult patients have received a transfusion at least once in their lifetimes [3]. Although transfusion improves disease severity and complications, severe iron overload is an inevitable complication, and chronically transfused iron-overloaded SCD patients have higher mortality than those with fewer transfusions and without iron overload [5].

Magnetic resonance imaging (MRI) is noninvasive, inexpensive, and widely available in developed countries [6]. Although serum ferritin is clinically used to estimate body iron stores, it reflects only around 1% of the total iron storage pool, and its measurement can be confounded by many conditions. In addition, liver iron content measured through MRI, which serves as a better indicator of whole-body iron, does not reflect heart iron loading [7]. The pancreatic iron burden may precede cardiac iron loading and is a powerful predictor of heart iron overload, and its early assessment and tailored chelation could prevent diabetes and preserve pancreatic reserve [8].

The primary purpose of this work was to quantitatively assess pancreatic iron loading in transfusion-dependent SCD patients. The secondary purposes were to assess pancreatic iron load in correlation to hepatic and cardiac iron load using MRI and to delineate the relationship between pancreatic iron load, clinical outcomes, and laboratory tests including serum ferritin and amylase.

## Materials and methods

### Patient population

This cross-sectional study included 66 children and young adults with SCD who were recruited as regular patients of the Pediatric Hematology Clinic, Children’s Hospital, a tertiary university hospital. Participation in the study was voluntary and required informed consent from the patients and/or their legal guardians. The study was approved by the institutional regulatory board of the Pediatric University Hospital. All procedures adhered to the ethical standards of the responsible committee on human experimentation (institutional and national) and with the Helsinki Declaration of 1975, as revised in 2008.

### Inclusion criteria

- Older than 5 years and able to perform MRI study.
- Patients with SCD who received packed RBCS blood transfusion more than 20 times in their lives showed an increased risk of iron overload. Thus, chelation therapy should be considered (according to National Cancer Comprehensive Network Clinical Practice Guidelines in Oncology [9]).

### Exclusion criteria

- Known to have contraindications for MRI, such as an implanted magnetic device, pacemaker, or claustrophobia.
- History of myocardial infarction, cardiac failure, or hepatic failure.
- Affliction with other transfusion-dependent diseases.

All recruited patients were subjected to detailed medical history review and full clinical examination with special emphasis on disease duration, anthropometric measures, cardiac disease, history of splenectomy, viral hepatic infection, and history of transfusion or chelation therapy. The transfusion that was received was calculated as transfusion index: volume of transfused packed red cells in ml per kg body weight per year.

Patients with SCD received monotherapy or combined chelation therapy. Mono-chelation included deferoxamine (DFO) infused subcutaneously in a dose that ranged from 30 to 45 mg/kg/day given 5 days/week, oral deferiprone (DFP) in a daily dose ranging from 50 to 100 mg/kg/day, or oral deferasirox (DFX) in a daily dose of 40 mg/kg/day. Assessment of patients’ compliance with chelation therapy involved reviewing patient self-reports, and the number of doses taken each day was checked using prescription refills and pill counts. A cutoff point below 80% was considered poor compliance to the regimen [10]. Hydroxyurea therapy was given orally in a dose of 20 mg/kg/day, with an increase to the maximum tolerated dose according to safety and response.

### Laboratory analysis

Peripheral venous blood samples were collected on potassium-ethylenediaminetetraacetic acid (K2-EDTA) for complete blood count (CBC) using Sysmex XT-1800i (Sysmex, Kobe, Japan) hemoglobin analysis by HPLC using D-10 (BioRad, Marnes La Coquette, France). To perform the chemical analysis and enzyme-linked immunosorbent assay (ELISA), clotted samples were obtained, and serum was separated by centrifugation for 15 min to perform liver function tests (including serum albumin, total bilirubin, alanine aminotransferase, aspartate aminotransferase, lactate dehydrogenase, and indirect bilirubin) using Cobas Integra 800 (Roche Diagnostics, Mannheim, Germany). Serum ferritin level was measured using the Immulite 1000 analyzer (Siemens Healthcare Diagnostics, Marburg, Germany) and accompanied by the calculation of the patient’s mean value of the year before the study to assess ferritin trend. As per relevant literature, the cutoff value of 2500 µg/L was used to classify patients into two groups as this has been defined as the best predictor of thalassemia complication [11].

### Magnetic resonance imaging (MRI) acquisition and image analysis

MRI examination was performed on a 1.5 Tesla superconductive MR Philips scanner (Achieva; Philips Medical Systems, Best, The Netherlands) in a tertiary university hospital without any contrast material. Patients were prepared and informed to remain motionless, avoid excessive swallowing, adjust respiration, and avoid several diaphragmatic motions. The duration of the study took approximately 10–15 min, and the system produced some loud noises.

(A) To complete quantitative measurement of pancreatic iron loading (R2*), the following steps were taken.

- Upper abdominal axial cuts were taken using a multi-echo gradient sequence at 12 simultaneous echo times (TE) with a field-of-view (FOV) span from the dome of the diaphragm to the inferior poles of the kidneys to ensure complete pancreatic coverage by 25 slices.
- The region of interest (ROI) was manually drawn over the pancreatic head or tail encompassing parenchymal tissue (mostly drawn over the pancreatic head) and took care to avoid confounding anatomy (large blood vessels or ducts) and areas with susceptibility artifacts from gastric or colic intraluminal gas. Then, the ROI was copied across all images.
- Grading of pancreatic iron loading (R2*): Normal: < 30 Hz, Mild: 30–100 Hz, Moderate: 100–400 Hz, and Severe: > 400 Hz [12].

(B) To complete quantitative measurement of myocardial T2*, the following steps were taken:

- Multi-echo turbo field echo (mTFE) cardiac black and white blood short-axis were obtained using ECG and respiratory-gated with a dedicated 12-element phased-array Torso coil using single 8–12 s breaths.
- The ROI was drawn in the interventricular septum encompassing both endocardial and epicardial regions.
- Grading of cardiac iron loading T2*: Normal > 20 ms, Mild: 15–20 ms, Moderate: 10–15 ms, and Severe < 10 ms [13].

(C) To quantitatively measure liver iron concentration (LIC), the following steps were taken:

- Upper abdominal axial cuts were taken using a multi-echo gradient sequence where the signal intensity of the liver parenchyma was acquired using region-based measurement.
- The ROI was placed over an axial mid-hepatic slice of the right hepatic lobe in an area free from vessels and bile ducts.
- Liver siderosis was measured using relaxation parameter T2*, and liver T2* values were then converted into R2* values (= 1000/T2*). Finally, LIC (mg/gdw) were calculated according to Garbowski et al.’s equation: LIC = 0.03 × R2* + 0.7 [14].
- Grading of liver iron loading LIC: Normal < 2 mg/g, Mild: 2–7 mg/g, Moderate: 7–15 mg/g, and Severe > 15 mg/g [15].

The pancreatic R* as well as myocardial and liver T2* were manually calculated via simple mathematical models by using Microsoft Excel Spread Sheet V3.0 [16]. The mean value of the signal intensity along different TE values was manually input into an Excel spreadsheet, and then, a curve-fitting truncation model consisting of a mono-exponential decay curve was applied [17].

(D) To qualitatively assess the renal iron overload:

- The upper abdominal axial cuts that were taken for coverage of the whole pancreatic tissue by 25 slices with a FOV spanning from the dome of the diaphragm to the inferior poles of the kidneys were used for qualitative assessment of the renal iron overload.
- The renal cortices contained the highest concentrations of glomeruli and proximal tubules, and the micro-anatomic locations contained the greatest iron deposition. Excess renal iron overload was determined by the presence of a hypointense signal of the renal cortex compared to the medulla on the T1-weighted images and accentuated reduction in cortical signal intensity on the T2-weighted images.

### Statistical analysis

The data were analyzed using Stata® version 14.2 (StataCorp LLC, College Station, TX, USA) and MedCalc© version 15.8 (MedCalc© Software bvba, Ostend, Belgium). Quantitative variables were described in the form of mean and standard deviation or median and interquartile range (IQR; 75th and 25th percentiles). Qualitative variables were described as numbers and percentages. The Kolmogorov–Smirnov test was used to test the distribution of normality. To compare parametric quantitative variables between two groups, Student’s *t* test was applied. To compare nonparametric quantitative variables between two groups, the Mann–Whitney test was used. Qualitative variables were compared using the chi-square (*x*^2^) test or Fischer’s exact test when frequencies were below five. Pearson correlation coefficients were used to assess the association between two normally distributed variables. When a variable was not normally distributed, a Spearman correlation test was performed. A *p* value < 0.05 was considered significant in all analyses.

## Results

This study included 66 patients (*n* = 66; 31 females and 35 males; 15.68 ± 7.02 years of age) with a history of SCD who had received repeated blood transfusions for cardiopulmonary complications and ACS (33.3%, 22 patients) as a secondary stroke preventive measure (13.7%, 9 patients) and for frequent sickling crisis and symptomatic anemia (53.0%, 35 patients).

Among the 66 patients, only 53 received chelation therapy. Of these 53 patients, 92.5% (49 patients) received monotherapy as follows: 35 patients (71.4%) received DFP, 13 patients (26.5%) received DFO, and only one patient (2.1%) received DFX. The remaining four patients (7.5%) required combined chelation therapy for the treatment of iron overload. Demographic, clinical, laboratory, and radiological characteristics of the studied patients with SCD are illustrated in Table 1.

**Table 1:** Characteristics of the studied patients with sickle cell disease

Variable	Sickle cell disease (*n* = 66)
Age (year); mean ± SD	15.68 ± 7.02
Male: female, *n* (%)	35 (53.0%): 31 (47.0%)
Positive family history of SCD, *n* (%)	43 (65.2%)
*Clinical characteristics*	
Splenectomized, *n* (%)	13 (20.0%)
Number of sickle crisis/year; median (IQR)	4 (2–8)
Sickle crisis ≥ 3/year, *n* (%)	35 (54.7%)
History of silent or manifest stroke, *n* (%)	9 (14.1%)
History of acute chest syndrome, *n* (%)	12 (18.8%)
Cardiopulmonary complications, *n* (%)	10 (15.6%)
Transfusion index (mL/kg/year)	120 (60–240)
Iron overload per day (mg/kg); mean ± SD	0.23 ± 0.15
On chelation therapy, *n* (%)	53 (80.3%)
Poor compliance to chelation, *n* (%)	21 (42.0%)
*Laboratory characteristics*	
Pre-transfusion hemoglobin (g/dL); mean ± SD	8.03 ± 1.42
HbS (%); mean ± SD	61.33 ± 20.70
HbF (%); median (IQR)	4.7 (1.3–12.2)
Serum amylase (U/L); mean ± SD	56.73 ± 21.43
Serum ferritin (ug/L); median (IQR)	2805 (median 940–4638)
Serum ferritin level > 2500; *n* (%)	33 (50%)
*Radiological characteristics*	
LIC (mg/g liver dry weight); median (IQR)	11.63 (5.81–20.31)
Normal; *n* (%)	7 (10.6%)
Mild; *n* (%)	16 (24.2%)
Moderate; *n* (%)	24 (36.4%)
Severe; *n* (%)	19 (28.8%)
Cardiac T2* (msec); mean ± SD	31.40 ± 6.58
Normal; *n* (%)	66 (100%)
Pancreatic MRI (msec); median (IQR)	53.80 (35.35–84.45)
Normal; *n* (%)	14 (21.2%)
Mild; *n* (%)	43 (65.1%)
Moderate; *n* (%)	8 (12.1%)
Severe; *n* (%)	1 (1.6%)

**Fig. 1:**
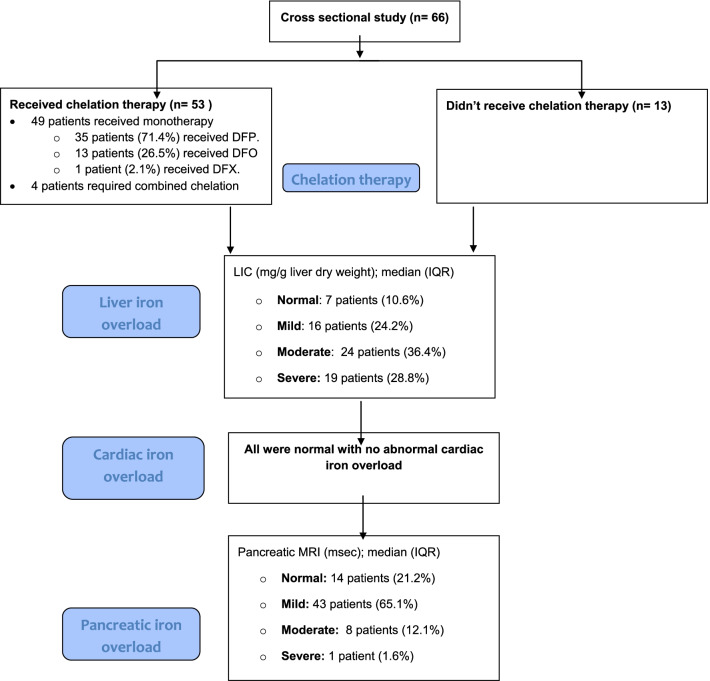
Flow diagram showing the number of patients under chelation therapy, as well as the hepatic, cardiac, and pancreatic iron loading among the sample population

**Fig. 2:**
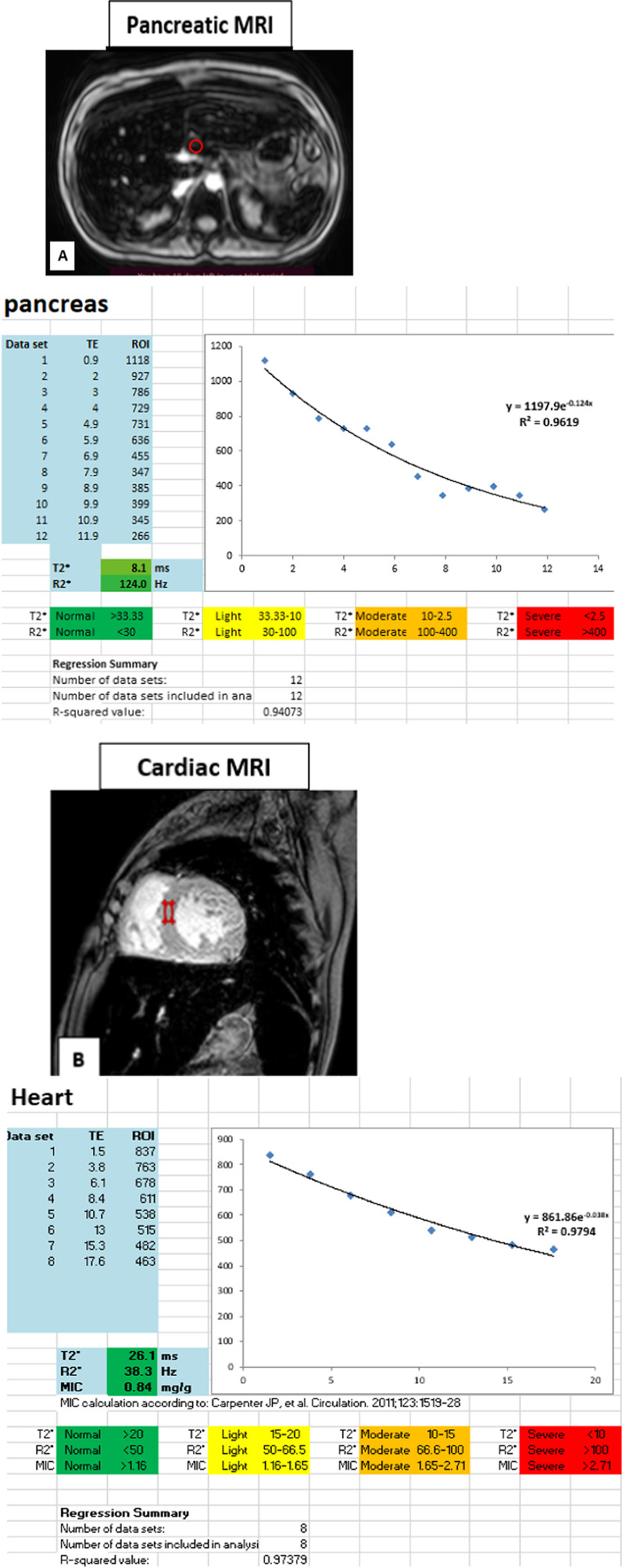

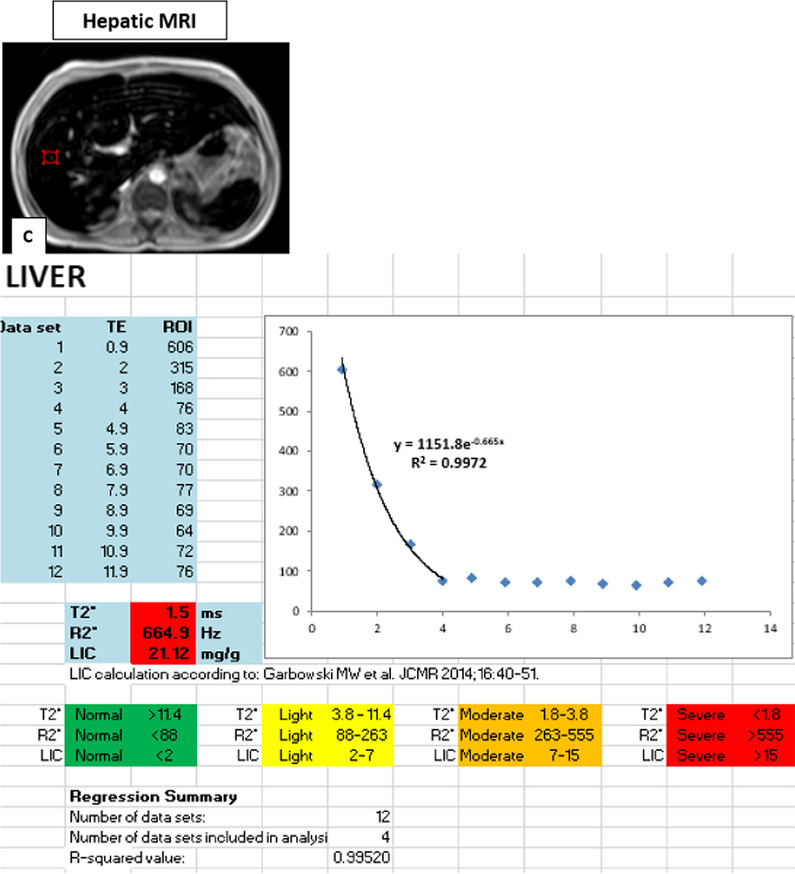
MRI of a transfusion-dependent patient with sickle cell disease using multiple echo times. **A** Axial MRI with a region of interest drawn in the head of the pancreas. **B** Left ventricular short-axis black blood MRI images sequence with a region of interest drawn at mid interventricular septum. **C** Axial MRI liver with a region of interest drawn in the periphery of the right lobe of the liver

Most of the patients (65.2%, 43 patients) demonstrated moderate-to-severe hepatic iron overload, 13.6% (9 patients) demonstrated moderate-to-severe iron overload within the pancreatic tissue, and none had a cardiac iron overload. Twenty-eight patients revealed a marked decrease in renal cortical signal intensity with almost sparing of the renal medulla (Figs. 1, 2).

To study the possible correlation between pancreatic iron overload in SCD patients, a comparison between SCD patients with normal pancreatic MRI and those with moderate-to-severe pancreatic MRI was performed, as illustrated in Table 2. The mild subgroup was omitted from this comparative study for two reasons. Firstly, the mild subgroup had a narrow zone of Hz of only 0–100 versus the moderate (100–400) and severe zones (more than 400 Hz). Secondly, the mild subgroup was in the gray zone between normal and significant iron overloading. Thus, adding this group with a relatively high percentage ratio (65.1%) of abnormal pancreatic MRI to the sample would have produced a great impact on the results.

**Table 2:** Comparison between sickle cell disease patients with normal and those with abnormal pancreatic MRI

Variable	Normal pancreatic MRI (*n* = 14)	Abnormal* pancreatic MRI (*n* = 9)	*p* value
Age (years); mean ± SD	17.79 ± 8.80	20.11 ± 8.80	0.543
Males; *n* (%)	8 (57.1)	3 (33.3)	0.265
Transfusion index (mL/kg/year); median (IQR)	240 (120–240)	120 (60–240)	0.158
Iron Overload (mg/kg/day); mean ± SD	0.30 ± 0.14	0.22 ± 0.17	0.226
On chelation; *n* (%)	11 (78.6)	7 (77.8)	0.964
Poor compliance to chelation; *n* (%)	5 (45.5)	5 (71.4)	0.280
Pre-transfusion hemoglobin (g/dL); mean ± SD	8.74 ± 1.64	6.61 ± 0.54	0.004
HbS (%); mean ± SD	67.79 ± 24.72	54.92 ± 25.69	0.312
HbF (%); median (IQR)	0 (0–8.2)	3.45 (3.1–12.2)	0.177
Lactate dehydrogenase (IU/L); mean ± SD	472.14 ± 155.57	768.38 ± 531.13	0.062
Total bilirubin (mg/dL); mean ± SD	2.42 ± 1.16	4.05 ± 1.59	0.012
Indirect bilirubin (mg/dL)	1.515 (0.96–2.1)	2.04 (1.89–2.71)	0.048
Serum amylase; mean ± SD	62.43 ± 24.15	70.00 ± 32.37	0.539
Serum ferritin (ug/L); median (IQR)	3670.5 (1456–4743)	1987 (1650–4313)	0.571
Serum ferritin level > 2500; *n* (%)	8 (57.1)	4 (44.4)	0.552
LIC (mg/g liver dry weight); median (IQR)	14.045 (7.35–20.83)	14.03 (9.2–24.89)	0.614
Normal; *n* (%)	1 (7.1)	0 (0.0)	0.859
Mild; *n* (%)	3 (21.4)	2 (22.2)	
Moderate; *n* (%)	5 (35.7)	3 (33.3)	
Severe; *n* (%)	5 (35.7)	4 (44.4)	
Cardiac T2* (msec); mean ± SD	31.51 ± 4.90	29.99 ± 3.87	0.442

*Patients with Abnormal^*^ pancreatic MRI include those with pancreatic MRI > 100 Hz

Patients who presented with a high level of hemolysis marker and a low level of pre-transfusion hemoglobin exhibited moderate-to-severe pancreatic MRI iron overload. Although the percentage of non-compliance to chelation therapy was higher (71.4%) in those who had abnormal pancreatic MRI than those with normal MRI (45.5%), the difference does not have statistical significance. To highlight the effect of iron overload, a comparison between SCD patients who had serum ferritin less than or equal to 2500 ug/L and those with high serum ferritin of more than 2500 ug/L was also performed, as illustrated in Table 3.

**Table 3:** Comparison between sickle cell disease patients who had serum ferritin ≤ and > 2500 ug/L

Variable	Serum ferritin ≤ 2500 ug/L (*n* = 33)	Serum ferritin > 2500 ug/L (*n* = 33)	*p* value
Age (years); mean ± SD	14.78 ± 6.85	16.55 ± 7.18	0.315
Males; *n* (%)	15 (46.9)	19 (57.6)	0.388
Iron Overload (mg/kg/day); mean ± SD	0.17 ± 0.14	0.28 ± 0.13	0.001
On chelation; *n* (%)	20 (62.5)	32 (97.0)	0.001
Poor compliance to chelation; *n* (%)	6 (31.6)	15 (50.0)	0.204
Pre-transfusion hemoglobin (g/dL); mean ± SD	7.66 ± 1.46	8.37 ± 1.30	0.046
HbS (%); mean ± SD	63.28 ± 16.04	59.57 ± 24.29	0.504
HbF (%); median (IQR)	6.2 (0.8–12.2)	3.6 (1.3–10)	0.579
Lactate dehydrogenase (IU/L); mean ± SD	547.38 ± 331.71	525.21 ± 204.67	0.749
Total bilirubin (mg/dL); mean ± SD	2.59 ± 1.50	2.60 ± 1.09	0.970
Indirect bilirubin (mg/dL)	1.33 (0.9–2.18)	1.8 (0.9–2.12)	0.549
Serum amylase; mean ± SD	57.41 ± 24.46	56.12 ± 18.74	0.815
LIC (mg/g liver dry weight); median (IQR)	6.19 (3.06–11.02)	16.16 (12.94–24.7)	0.000
Normal; *n* (%)	7 (21.9)	0 (0.0)	0.000
Mild; *n* (%)	13 (40.6)	2 (6.1)	
Moderate; *n* (%)	10 (31.3)	14 (42.4)	
Severe; *n* (%)	2 (6.3)	17 (51.5)	
Cardiac T2* (msec); mean ± SD	32.03 ± 5.09	30.79 ± 7.80	0.454
Pancreatic MRI (msec); median (IQR)	52.85 (35.35 – 83.5)	54.9 (31.8 – 87)	0.847

A correlation study of pancreatic MRI among the SCD patients revealed that there was a non-significant negative correlation between pancreatic MRI and transfusion index (*p* = 0.314), iron overload per day (*p* = 0.424), pre-transfusion hemoglobin (*p* = 0.051), serum amylase (*p* = 0.730), HbS% (*p* = 0.663), and serum ferritin (*p* = 0.964). In addition, there was a non-significant positive correlation with LIC (*p* = 0.069). Furthermore, there was no significant correlation between serum amylase and other studied parameters.

## Discussion

Transfusion is used in patients with SCD to increase blood’s oxygen-carrying capacity and to improve blood flow [4]. The recruited children and young adults with SCD were a unique population who received frequent transfusions as prophylaxis and as therapy for major complications of SCD. However, iron overload is an unavoidable complication of transfusions [4]; consequently, the studied SCD patients presented high iron overload/day with an estimated average value of 0.23 ± 0.15 mg/kg, which leads to iron accumulation. Fortunately, 80.3% of the studied patients received monotherapy chelation.

MRI does not image iron directly; it images water protons diffusing near iron deposits [6], which causes local distortion in the magnetic field inhomogeneity (T2^∗^) and loss of signal intensity in proportion to its deposition [18]. MRI represents a safe, noninvasive, highly reproducible modality [19, 20] that provides new insights into the dynamics of iron overload [21].

Iron causes MRI images to darken at a rate proportional to the hepatic iron load, with the half-life of this darkening defined as T2*. The rate of darkening, designated as R2*, is the reciprocal of T2* and is proportional to the iron content of the tissues. MRI scanning estimates tissue iron concentration both by gradient echo imaging, which provides T2*, and spin echo imaging, which provides T2, the reciprocal of R2 [22].

R2 and R2* methods have respective theoretical advantages and disadvantages. R2 techniques are insensitive to the size and shape of the imaging “voxel” as well as external magnetic inhomogeneities, while R2* methods can be influenced by these factors. In contrast, R2* measurements are more robust to variations in the length scale of iron deposition and can accurately reflect the bulk magnetic susceptibility of tissues. R2* measurements can also be performed in a single breath-hold, while R2 methods take 5 to 20 min (depending on technique). R2* measurements are robust to long-range magnetic disturbances; thus, one would expect a linear relationship between R2* and iron over the entire physiologic range of iron deposition [23, 24].

There are two basic types of pulse sequences: the spin echo (SE) and the gradient echo (GRE). To measure signal intensity and quantify iron concentration, GRE T2* and SE T2 sequences are used. The GRE sequence generates a T2* decay curve, which is much faster and very sensitive even to small amounts of iron deposition. In contrast, the SE sequence generates a T2 decay curve, which is a more time-consuming process [25].

In the current study, MRI T2* relaxometry method was used to concurrently quantify hepatic, myocardial, and pancreatic iron in the same setting with short acquisition times and fast scanning through the multi-echo sequence, which is particularly beneficial in the pediatric population. A range of echo times was used to allow accurate quantification of T2* values in cases of severe iron overload and to provide suitable sensitivity at low tissue iron levels. The use of constant repetition time between all echo times eliminated any T1 effects that might skew the data when using the conventional sequence [26].

There is no definitive gold standard for T2* post-processing [15]. Consequently, iron content was calculated in the current study through a relatively inexpensive, commercially available Excel spreadsheet with a linear mono-exponential fitting model that is reported to have a slightly higher coefficient of variation compared with the nonlinear fitting used in CMR tools [16]. This Excel-based approach would be the most accessible program for most physicians, especially in limited-resource settings, and avoids the complicated extra technical step and costs of licensing the necessary complementary bases.

The liver is the dominant storage organ for excess iron acquisition and mobilization of iron in response to iron chelation [27]. An LIC of more than seven milligrams Fe/gram dry liver weight represents the best threshold for determining the presence of hepatic fibrosis [27] and vascular morbidity [28]. The majority of patients (65.2%) had moderate-to-severe liver iron overload, confirming the previously reported finding that liver toxicity in SCD occurs at similar levels to those observed in patients with thalassemia major (TM) [28–30].

The heart, in contrast to the liver, has robust mechanisms to prevent excessive transferrin-mediated uptake [27]. The studied children and young adults exhibited moderate-to-severe hepatic iron loading, with no evidence of cardiac iron loading endorsing that chronically transfused patients with SCD had a lower risk of cardiac complication in comparison with patients with TM [31]. Delayed cardiac iron uptake compared to many other extrahepatic organs including the pancreas [32] confirms that iron overload selectively targets the liver in patients with SCA, initially relatively sparing the heart [33]. However, the heart becomes vulnerable to iron loading once the “threshold” LICs are reached, and that threshold is higher in SCD [34] (15–20 mg/g dry weight) than in TM [35].

Pancreatic iron overload can impair the exocrine and endocrine functions of the pancreas [8], which, unlike the liver, may not regenerate or remodel even with the reduction in hemosiderosis [36]. This necessitates early assessment of pancreas iron and tailored chelation that may prevent diabetes and preserve pancreatic reserve [8]. Most of the recruited chronically transfused SCD patients (86%) had normal-to-mild pancreatic iron overload, confirming that they are less likely to develop pancreatic iron overload compared to patients with TM; this is likely because iron released by transfusion and hemolysis is efficiently handled by effective erythropoiesis [37], thus keeping transferrin saturations [38] and non-transferrin-bound iron (NTBI) levels low [39]. Furthermore, SCD patients have shorter and less intense transfusion exposure [40] even when aggressive chronic transfusion therapy is used as it is often started later in life and at a lower intensity [41]. In line with these points, Noetzli et al. found that chronically transfused SCD patients are less likely to develop moderate-to-severe pancreatic iron overload even after correcting for differences in transfusion duration, transfusion intensity, and severity of iron loading [42].

SCD patients with a history of low pre-transfusion hemoglobin levels and high levels of hemolysis markers revealed moderate-to-severe pancreatic iron MRI overload, thereby supporting the hypothetical relationship between hemolysis and pancreatic iron overload. Pancreatic iron burden precedes and is a powerful predictor of heart iron overload [8] as both organs have the same L-type calcium iron channels [36]. In this study, pancreatic R2* did not correlate with cardiac T2^*^ as all patients had normal cardiac T2*, and it had a nonlinear relationship with LIC. This data suggests that heavy hepatic siderosis is a prerequisite for cardiac and endocrine siderosis in SCD, unlike in TM [43], and that pancreas R2* values probably represent the most viable surrogate index for extrahepatic risk [44].

The renal cortices contain the highest concentrations of glomeruli and proximal tubules, and it is the micro-anatomic locations that contain the greatest iron deposition in SCD patients [45]; this is consistent with the finding that nearly half of patients revealed a marked decrease in renal cortical signal intensity (which represents iron loading) with almost sparing of the renal medulla. In the current study, renal iron was not quantitatively assessed, and further studies are needed to assess kidney iron burden in patients with SCD.

## Conclusion

Chronically transfused patients with SCD have a high frequency of iron overload complications including pancreatic iron deposition, thus necessitating proper monitoring of the overall body iron balance as well as detection of extrahepatic iron deposition.

### Study limitation

Contributions from multicenter will be of additive value to better assess such important complications of extrahepatic iron deposition. ROI positioning in the pancreatic tissue is sometimes complicated due to tissue inhomogeneities and breathing artifacts. Additionally, the pancreas may be difficult to locate in older, splenectomized subjects because of glandular apoptosis, fatty replacement, and loss of normal anatomic landmarks. Moreover, the surrounding confounding anatomy (e.g., large blood vessels or ducts) and areas involved in susceptibility artifacts from gastric or colic intraluminal gas also hinder proper pancreatic assessment and may hamper the results. The effect of iron overload upon pancreatic functioning, and especially the endocrine function, needs to be evaluated to predict the risk of diabetes mellitus among transfusion-dependent SCD patients. Renal iron was not quantitatively assessed, and further studies need to be conducted to assess kidney iron burden in patients with SCD.

## Data Availability

Available on request with the corresponding author. The authors declare that they had full access to all of the data in this study, and the authors take complete responsibility for the integrity of the data and the accuracy of the data analysis.

## Abbreviations

ACS: Acute chest syndrome
ALT: Alanine aminotransferase
AST: Aspartate aminotransferase
CBC: Complete blood count
FOV: Field of view
HbS: Sickle hemoglobin
LDH: Lactate dehydrogenase
LIC: Liver iron concentration
MRI: Magnetic resonance imaging
ms: Milliseconds
mTFE: Multi-echo turbo field echo
ROI: Region of interest
SCD: Sickle cell disease
TE: Echo time
TM: Thalassemia major
